# Opposing Roles of IGFBP-3 and Heparanase in Regulating A549 Lung Cancer Cell Survival

**DOI:** 10.3390/cells11223533

**Published:** 2022-11-08

**Authors:** Hind Al Khashali, Jadziah Wareham, Ravel Ray, Ben Haddad, Kai-Ling Coleman, Robert Ranzenberger, Patrick McCombs, Jeffrey Guthrie, Deborah Heyl, Hedeel Guy Evans

**Affiliations:** Chemistry Department, Eastern Michigan University, Ypsilanti, MI 48197, USA

**Keywords:** heparanase, IGFBP-3, lung cancer, heparan sulfate, hyaluronan, CD44, p53, signaling, extracellular

## Abstract

In this study, we examined the roles of heparanase and IGFBP-3 in regulating A549 and H1299 non-small-cell lung cancer (NSCLC) survival. We found that H1299 cells, known to be p53-null with no expression of IGFBP-3, had higher heparanase levels and activity and higher levels of heparan sulfate (HS) in the media compared to the media of A549 cells. Inhibiting heparanase activity or its expression using siRNA had no effect on the levels of IGFBP-3 in the media of A549 cells, reduced the levels of soluble HS fragments, and led to decreased interactions between IGFBP-3 and HS in the media. HS competed with HA for binding to IGFBP-3 or IGFBP-3 peptide (^215^-KKGFYKKKQCRPSKGRKR-^232^) but not the mutant peptide (K228AR230A). HS abolished the cytotoxic effects of IGFBP-3 but not upon blocking HA–CD44 signaling with the anti-CD44 antibody (5F12). Blocking HA–CD44 signaling decreased the levels of heparanase in the media of both A549 and H1299 cell lines and increased p53 activity and the levels of IGFBP-3 in A549 cell media. Knockdown of p53 led to increased heparanase levels and reduced IGFBP-3 levels in A549 cell media while knockdown of IGFBP-3 in A549 cells blocked p53 activity and increased heparanase levels in the media.

## 1. Introduction

Non-small-cell lung carcinoma (NSCLC) accounts for ~85% of all lung cancers and, compared to small-cell carcinoma, is relatively insensitive to chemotherapy [1]. While several advancements have been made over the last two decades in our understanding of the molecular mechanisms behind tumor progression, the overall rate of survival for NSCLC remains low [1,2]. Therefore, continued research efforts aimed at understanding these molecular mechanisms are required to improve outcomes in NSCLC [1,2].

Glycosaminoglycans (GAGs) are linear, long, acidic carbohydrate polymers composed of repeating, negatively charged, hydrophilic disaccharide units [3]. Heparan sulfate (HS) is an unbranched biopolymer and member of the GAG family that consists of repeating negatively charged sulfated disaccharide units of uronic acid (glucuronic acid or iduronic acid), 1,4 linked to glucosamine [4,5]. It is present in all cell types and is widely abundant in human tissue [4,6]. It plays a role in oncogenesis and numerous other cellular processes by non-covalently interacting with key growth factors, receptors, and other type of proteins, influencing proliferation and modulation of the microenvironment [4,7].

HS chains are covalently bound to a protein core to form HS-proteoglycans (HSPGs) which are predominantly found on the cell surface and in the extracellular matrix (ECM) [5,6,8]. HSPGs are known to be involved in storage of growth factors, ECM assembly, cell signaling, and cell adhesion [4,5].

Heparanase is an endo-β-D-glucuronidase that acts both at the cell-surface and within the ECM to cleave polymeric HS sidechains into shorter chain-length oligosaccharides, releasing bioactive HS fragments from the ECM [9,10]. These discrete biologically active fragments are still able to bind HS-binding proteins [11,12]. Despite the central role of HSPGs, heparanase is the only known enzyme encoded in the mammalian genome that cleaves HS specifically at intrachain sites [6]. Heparanase is known to be more expressed in tumors and in NSCLC compared to normal tissues and to be enzymatically active; heparanase, expressed as an enzymatically inactive 65 kDa precursor polypeptide, needs to be cleaved to yield a heterodimer of 8 and 50 kDa subunits [6,10,11]. Numerous malignancies and poor prognosis have been associated with augmented expression levels of heparanase [10]. Heparanase plays important roles in tumor growth, correlates with the metastatic potential of cancer cells and, as such, has generated interest as a potential target against tumor development [4,6,10,12]. Release of HS side chains is thought to increase the pro-tumorigenic effects of heparanase promoting tumor invasion [10]. Preclinical trials have shown that blocking heparanase with inhibitors, developed as antineoplastic agents, significantly diminishes tumor growth and metastasis [6,10,12]. The function of heparanase at the cell surface, however, can also be non-enzymatic, HS-independent, and not limited to its ability to cleave HS or to the release of proteins or growth factors that have been sequestered [11].

Under normal physiological conditions, ECM dynamics are tightly regulated [13]. When this regulation is corrupted, ECM dynamics become deregulated, resulting in loss of cellular signaling and various human diseases, including cancer [13,14]. A major component of the ECM is hyaluronan (HA), an anionic GAG polymer without any known post-synthetic modification, composed of a simple disaccharide sequence (D-glucuronic acid and D-N-acetylglucosamine), and lacking a covalently bound protein core [15,16,17,18]. HA is non-sulfated, in contrast with other GAGs, and therefore has the least net negative charge [15,19,20]. HA is synthesized by transmembrane proteins at the intracellular surface of the plasma membrane, and the growing polymer is simultaneously extruded into the extracellular space [19]. HA is largely abundant extracellularly and known to accumulate to high levels in lung adenocarcinomas [19,21,22]. Via interactions with its binding proteins, HA is associated with rapid ECM remodeling affecting disease progression [16,19,23]. HA binding to its main receptor, CD44, has been shown to promote cell growth and survival pathways [24]. Perturbation of HA–CD44 signaling by, for example, using soluble CD44 or monoclonal CD44 antibodies, has been reported to block cell survival [16,21,25,26].

The mature IGFBP-3 protein is 264-amino acid residues long after cleavage of the 27-residue signal peptide and is predominantly secreted [27,28]. The protein is multifunctional and plays diverse roles in both the intra- and extra-cellular environment [28,29,30]. IGFBP-3 belongs to a family of six IGF binding proteins that share highly conserved structures [27,28]. Expression of IGFBP-3 induces apoptosis and inhibits the proliferation of NSCLC cells [31,32,33,34]. This expression is lost in lung cancer [35] and associated with poor diagnosis in stage I NSCLC patients [31,36,37,38,39]. IGFBP-3 has been shown to have an inverse correlation with the risk of lung cancer [34]. The protein is known to exhibit antitumor activities in lung carcinoma among various other solid tumor models [40,41], and to block NSCLC cell growth and survival [33,42]. IGFBP-3 overexpression suppressed the growth and metastatic activities of NSCLC cells by potently inducing apoptosis [33,43,44,45].

Mature human IGFBP-3 is composed of three structural domains (N-terminal domain, mid-region, and C-terminal domain) [28]. The C-terminal domain has an 18-basic amino acid motif defined by amino acid residues 215–232 of mature IGFBP-3 previously shown to bind GAGs, including heparin, HS, and HA [27,28,46,47,48]. It was shown earlier that the linear, polyanionic, sulfated GAG heparin, known to possess anti-cancer properties, competes for IGFBP-3-binding to cell surface HSPGs resulting in increased IGFBP-3 accumulation in the media [49]. Earlier, we published that IGFBP-3 binds HA through this 18-residue basic motif and blocks HA interactions with its receptor, CD44, reducing viability of A549 human lung cancer cells [50,51,52].

The purpose of this study was to examine whether the levels of IGFBP-3 and heparanase are regulated by disruption of HA–CD44 signaling by IGFBP-3 in the extracellular milieu via a p53-dependent manner. We found increased IGFBP-3 levels and decreased heparanase levels and activity upon blocking HA–CD44 signaling and p53 activation. Decreased heparanase levels led to diminished soluble HS fragments and formation of the IGFBP-3-HS complex allowing further disruption of HA–CD44 signaling by IGFBP-3 not bound to HS, decreasing cell survival.

## 2. Materials and Methods

### 2.1. Materials

Most of the material used in this study was purchased as we reported earlier [50,51,52,53,54]. Phosphate-Buffered Saline (PBS), nitrocellulose membranes, streptavidin-horseradish peroxidase (HRP) conjugate, Ponceau S solution, heparan sulfate, phenylmethylsulfonyl fluoride (PMSF), and biotin-hyaluronan (B1557) were purchased from Sigma-Aldrich (Burlington, MA, USA). The IGFBP-3 protein (10430-H07H) was purchased from SinoBiological (Wayne, PA, USA). The heparanase inhibitor OGT 2115 was obtained from R&D Systems (Minneapolis, MN, USA). Heparanase siRNA was obtained from MyBioSource (San Diego, CA, USA). CD44 antibody (5F12) (MA5-12394), mouse α-tubulin monoclonal antibody (DM1A), goat anti-rabbit IgG (H + L) secondary antibody (HRP, 31466), 3,3′,5,5′-tetramethylbenzidine, Lipofectamine 2000 Transfection Reagent, the Halt Protease and Phosphatase Inhibitor Cocktail, the BCA protein assay kit, and the SuperSignal West Pico luminol (chemiluminescence) reagent were obtained from ThermoFisher (Waltham, MA, USA). Donkey anti-mouse IgG (HRP) (ab205724), and rabbit anti-goat IgG H&L (HRP) (ab6741) were purchased from Abcam (Waltham, MA, USA). The m-IgGκ BP-HRP was obtained from Santa Cruz Biotechnology (Dallas, TX, USA). SignalSilence p53 siRNA I (6231), SignalSilence Control siRNA (Unconjugated, 6568), and p53 antibody (9282) were purchased from Cell Signaling Technology (Danvers, MA, USA).

### 2.2. Cell Culture

As previously reported [50,51,52,53,54,55,56,57,58], 25 cm^2^ tissue culture flasks were seeded with either human A549 (ATCC CCL-185) or H1299 (ATCC CRL-5803) NSCLC cells (ATCC, Manassas, VA, USA) in 5 mL of HyClone DMEM/F12 medium (GE Healthcare Life Sciences, Pittsburgh, PA, USA) supplemented with 10% Fetalgro BGS (RMBIO, Missoula, MT, USA) and 50 U/mL penicillin-streptomycin (Invitrogen Life Technologies, Carlsbad, CA, USA). Cells were grown overnight in an incubator set to 37 °C, 95% humidity, and 5% CO_2_. A hemocytometer was used to count the cells after staining with trypan blue. The doubling time for H1299 cells was 22.5 ± 4.5 h, while that for A549 cells was 23.5 ± 3.5 h.

### 2.3. Solid Phase Peptide Synthesis and Purification

We synthesized the 18-amino acid residue (^215^-KKGFYKKKQCRPSKGRKR-^232^) heparin-binding domain of IGFBP-3 in the C-terminal region of the protein [59] that also binds HA along with the IGFBP-3 (^215^-KKGFYKKKQCRPSAGAKR-^232^) mutant peptide (K228AR230A), as a negative control, since we previously showed that it completely lacks the ability to bind HA or block HA–CD44 signaling [51,52]. Fluorenylmethyloxycarbonyl (Fmoc) protected L-amino acids and *O*-benzotriazolyl-*N*,*N*,*N*′,*N*′-tetramethyluronium hexafluorophosphate (HBTU), used to synthesize the peptides in this study, were purchased from Anaspec Inc (Fremont, CA, USA). and Combi-Blocks (San Diego, CA, USA). Dichloromethane (DCM) was purchased from Acros Organics. Dimethylformamide (DMF) and HPLC-grade acetonitrile (ACN) were obtained from VWR. Piperidine, triisopropylsilane (TIS), diethyl ether, ethanol, phenol, and trifluoroacetic acid (TFA) were purchased from Sigma-Aldrich. Rink amide MBHA resin was purchased from Nova Biochem (San Diego, CA, USA).

Peptides were synthesized as described previously [51,52] on a 0.1 mmole scale on methylbenzhydrylamine (MBHA) resin using a Protein Technologies PS3 peptide synthesizer. RP-HPLC was used to purify the peptides using a Phenomenex C18 column (25 cm × 2.2 cm), with a solvent system of 0.1% TFA in water (solvent A) and 0.1% TFA in ACN (solvent B), with a 2 h gradient of 10 to 50% solvent B at 10 mL/min. Final purity was determined at 220 nm by analytical RP-HPLC using a Phenomenex C18 column (25 cm × 4.6 mm). Peptide molecular weights were confirmed by paper spray ionization mass spectrometry. For assays, peptides were dissolved in 1% DMSO in pH 7.4 PBS buffer, to a final concentration of 1 mg/mL.

### 2.4. ELISA

ELISAs were conducted, as we reported previously, using Nunc MaxiSorp 96-well Flat-Bottom plate (ThermoFisher) wells [50,51,54,57,60]. All absorbance measurements were in the linear range. To monitor non-specific binding, negative control wells on the plates included, for example, bound pure IGFBP-3 protein or peptide, then adding all components, streptavidin-horseradish peroxidase and TMB, but without addition of biotin-HA. Before analysis, the OD from the data were corrected for non-specific binding by subtracting the mean background absorbance for the negative controls. Statistical analysis was determined by the GraphPad Prism 9.4.1 software. Data were expressed as the mean ± S.D. Three to five independent experiments were carried out in triplicate for each assay condition.

### 2.5. MTT Assay

The MTT reduction assay (Sigma-Aldrich), used to measure cell viability, was carried out as we reported earlier [50,51,61]. The absorbance was measured at 570 nm in a plate reader. All absorbance measurements were in the linear range. Untreated cells or wells containing only DMSO and media were used as a positive and negative control, respectively.

### 2.6. Quantitation of Heparanase Levels

The levels of heparanase were quantitated using the human heparanase sandwich ELISA kit (ab256401) following the manufacturer’s protocol. Briefly, the method employs a capture antibody conjugated to an affinity tag and a detector antibody conjugated to a HRP reporter. The complex including the capture antibody, the analyte, and detector antibody is then recognized by a monoclonal antibody used to coat the plates. Samples were first added to the wells, followed by addition of the antibody mix. Following the incubation, the wells were washed, then the TMB development solution was added resulting in a blue color. The reaction was then stopped by addition of the stop solution converting the color from blue to yellow and the intensity measured at 450 nm.

### 2.7. Heparanase Activity Assay

The activity of heparanase was measured using the AMSBIO kit (Ra001-BE-K) following the instructions provided by the manufacturer. Briefly, wells are covalently coated with biotinylated-HS. The media of cells treated as indicated was then added to the wells and allowed to incubate overnight at 37 °C. The HS fragments were then washed away followed by the addition of HRP-conjugated streptavidin and the TMB substrate. The decrease in the absorbance is directly proportional to heparanase activity.

### 2.8. HS Quantitation Assay

The levels of HS were quantitated using the Aviva Systems Biology HS ELISA Kit (OKEH02552) according to the instructions provided by the manufacturer. Briefly, an HS-specific antibody is pre-coated onto a 96-well plate. Samples were added to the wells, followed by addition of a biotinylated detector antibody specific for HS. After washing the wells, avidin-peroxidase conjugate was then added. After washing away the unbound conjugate, the TMB substrate was added and catalyzed by HRP resulting in a blue color product that changed to yellow following the addition of an acidic stop solution. The absorbance was measured at 450 nm and was quantitatively proportional to the amount of HS captured in the wells.

### 2.9. Quantitation of IGFBP-3 Levels

The levels of IGFBP-3 were quantitated using the human IGFBP-3 (EHIGFBP3, ThermoFisher) ELISA kit. In this assay, the amount of IGFBP-3 bound between a matched pair of antibodies is measured. In brief, samples were added to wells precoated with an IGFBP-3-specific antibody. After addition of the second detector antibody conjugated to biotin, Streptavidin-HRP, and a TMB substrate solution, a signal was developed, and the absorbance measured at 450 nm. The signal was directly proportional to the concentration of IGFBP-3 in the sample.

### 2.10. p53 Transcription Factor Activity Assay

The activity of p53 was assayed using the colorimetric BioVision’s p53 transcription factor activity assay (Catalog # K923-100) kit. Briefly, a 96-well plate is coated with double stranded oligonucleotides. Cell lysates containing activated p53 were then added to the wells allowing interaction with the oligonucleotides in the plate wells. A p53 primary antibody was then added followed by addition of a HRP-conjugated secondary antibody. The color signal was developed after addition of the TMB substrate and measured at 450 nm.

### 2.11. Western Blotting

Samples of the media or cell lysate supernatants collected as indicated were analyzed according to our previous protocols [50,51,56]. Briefly, attached live cells were harvested and the cell pellet was resuspended in 1 mL lysis buffer consisting of 20 mM Tris/HCl, pH 7.5, 137 mM NaCl, 1% triton X-100, 10% glycerol, 1 mM PMSF, and Halt protease and phosphatase inhibitor cocktail (ThermoFisher). Samples were briefly sonicated, centrifuged and the supernatants were stored at −80 °C. Media samples were centrifuged, and the supernatants were stored at −80°C until further analysis. The BCA protein assay kit was used to measure the protein concentration. Following the methods that we reported previously [50], samples were fractionated by SDS-PAGE on a 12% gel then transferred to a nitrocellulose membrane. The membrane was blocked in TBST buffer, pH 7.6, containing 5% nonfat milk for 6 h at 4 °C, incubated with the primary and secondary antibodies, developed using SuperSignal West Pico luminol (chemiluminescence) reagent, and imaged with a Bio-Rad molecular imager.

### 2.12. SiRNA Transfection

Transfections were carried out according to our methods reported earlier [51,62]. The day before transfection, cells were seeded at a density of 2 × 10^4^ cells in 25 cm^2^ flasks. Control siRNA, p53 siRNA, IGFBP-3 siRNA, or heparanase siRNA, were each mixed with Lipofectamine 2000 transfection reagent diluted in Opti-MEM Media (ThermoFisher) as recommended by the manufacturer, then the mixtures were added to the cells at a final concentration of 100 nM for each siRNA. The cells were then incubated at 37 °C for 12 h followed by the specific treatments as indicated. Cells exposed to Lipofectamine 2000 alone were used as a mock control. The media was used to quantitate the levels of heparanase and IGFBP-3 and the cell lysate supernatant was used to measure the activity of p53, as described above. Each measurement represents the mean ± S.D. of three–five independent experiments, each performed in triplicate.

### 2.13. Statistical Analysis

The analysis was carried out as we previously reported [51,52,53,54,56]. Each experiment in this study was performed at least in triplicate and repeated a minimum of three times. Statistical values are expressed as the mean ± Standard Deviation (SD). To evaluate the statistical differences, the Mann–Whitney test and a non-parametric Kruskal–Wallis test were performed. All the statistical tests were two-sided and a *p* value of <0.05 was considered statistically significant in all cases. GraphPad Prism (GraphPad Software, 9.4.1) was used for the statistical analysis.

## 3. Results

### 3.1. H1299 Cell Media Had Higher Heparanase Levels and Activity and Higher Levels of HS Than the Media of A549 Cells

**Figure 1:**
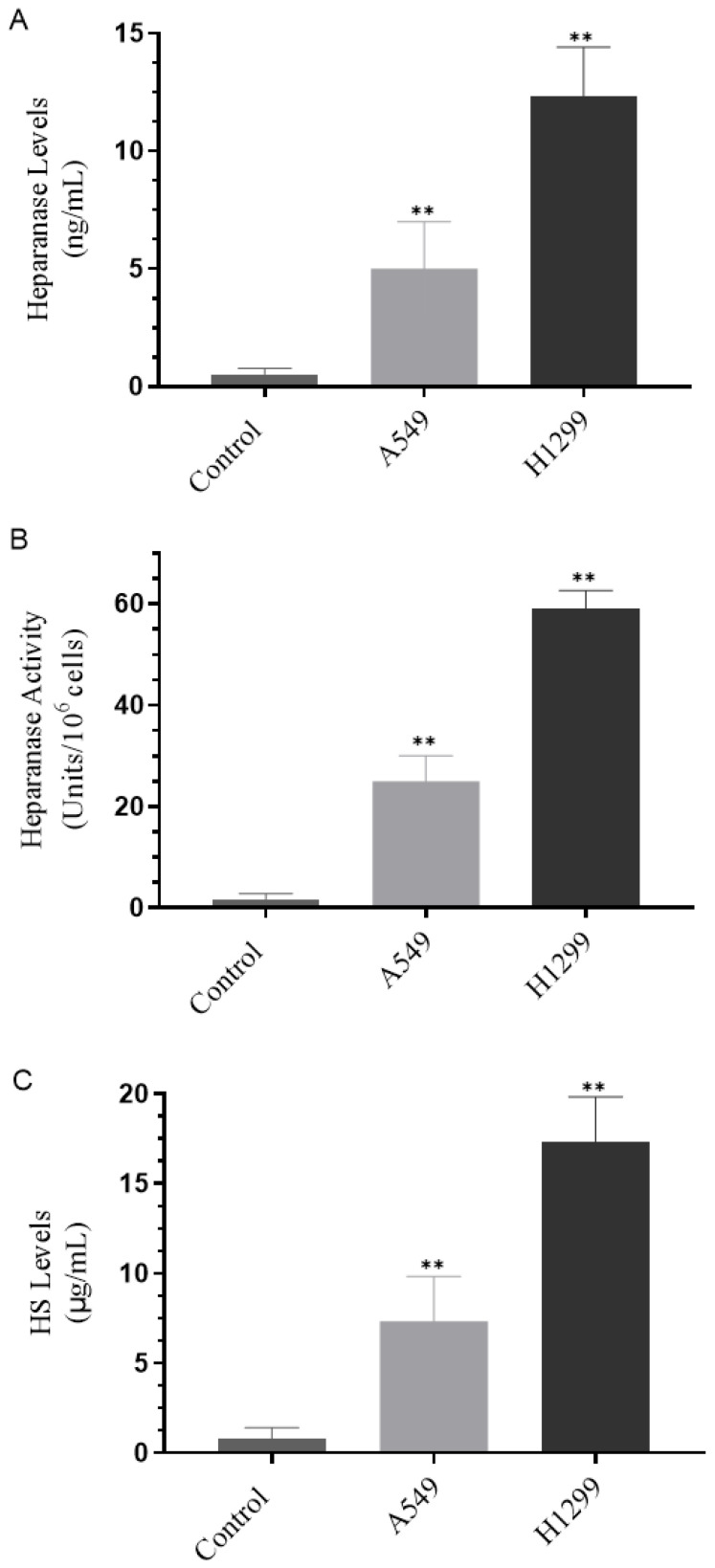
Higher heparanase levels and activity and higher levels of HS are found in H1299 cell media than in the media of A549 cells. Cells (0.2 × 10^5^) were grown in 10% FBS-supplemented media for 24 h then serum-starved overnight. The cell monolayers were then incubated in serum-free media for 72 h then the levels of heparanase (**A**) and activity (**B**) along with the levels of HS (**C**) were measured as described in the Materials and Methods section. Data were processed using the GraphPad Prism 9.4.1 software and presented as the mean ± S.D. of three independent assays, each performed in triplicate. Asterisks indicate a statistically significant difference from the control using media not incubated with cells. Mann–Whitney test, ** *p* < 0.01.

The tumor suppressor, p53, is known to directly bind the heparanase gene promoter suppressing its transcription while, conversely, an increase in heparanase gene expression and enzymatic activity was found upon elimination or inhibition of p53 in several cell types [10,63]. To examine the levels of heparanase and activity and the levels of HS in NSCLC, we used two human NSCLC cell lines [35], A549 (p53-positive), and H1299 (p53-null) [64]. Cells (0.2 × 10^5^) were grown in 10% FBS-supplemented media for 24 h then serum-starved overnight. The cell monolayers were then incubated in serum-free media for 72 h then the levels of heparanase (Figure 1A) and activity (Figure 1B), along with the levels of HS (Figure 1C) were measured as described in the Materials and Methods section.

The levels (Figure 1A) of heparanase were lower in A549 cell media (~5 ng/mL) than in H1299 cell media (~12.5 ng/mL). These levels correlated with the heparanase activity (Figure 1B) (~25- and 60-units/10^6^ cells in A549 and H1299 cell media, respectively). Similarly, the HS concentration was higher in the media of H1299 cells (~17.5 µg/mL) as compared to that found in A549 cell media (~7.5 µg/mL) (Figure 1C).

### 3.2. Inhibiting Heparanase Activity or Its Expression Using siRNA Led to Decreased HS Level in the Media and Reduced the Amount of IGFBP-3 Bound to HS

**Figure 2:**
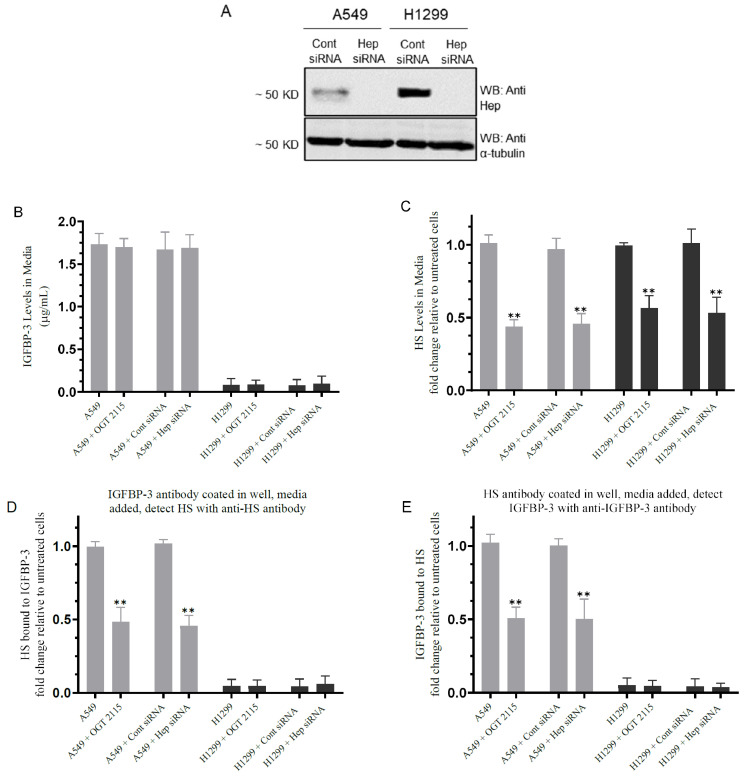
Blocking heparanase activity or expression using siRNA decreased HS level in the media and the amount of IGFBP-3 bound to HS. Cells (0.2 × 10^5^) were grown in 10% FBS-supplemented media for 24 h. The following day, the cell monolayers were incubated in serum-free media for 24 h, then treated as indicated for 72 h with OGT 2115 (100 µM) or with siRNA as described in the Methods section. The same concentration of total protein (15 µL of 600 µg/mL) of the cell lysates (**A**) was used for Western blotting using the indicated antibodies. As a loading control, anti α-tubulin antibodies were used. The levels of IGFBP-3 (**B**,**E**) and HS (**C**,**D**) were measured on the same amount of protein (3 µL of 600 µg/mL total protein) in the media as described in the Materials and Methods section. The graphs summarize the results expressed as means ± SD (*n* = 5) using the GraphPad 9.4.1 software. Asterisks indicate a statistically significant difference from the corresponding samples without inhibitor treatment or those treated with control siRNA of each cell line as indicated, Mann–Whitney test. The absence of asterisks indicates no significance, ** *p* < 0.0l.

Mature IGFBP-3 is known to associate with GAGs including HS via its 18-basic amino acid motif in the C-terminal domain (^215^-KKGFYKKKQCRPSKGRKR-^232^) [27,28,46,47,48]. To examine the effect of blocking heparanase activity or expression on the levels of IGFBP-3 and HS in the media and on the binding of IGFBP-3 to HS, cells were grown in 10% FBS-supplemented media for 24 h then incubated in serum-free media overnight. The cells were then treated as indicated with OGT 2115 or with siRNA as described in the Materials and Methods section (Figure 2).

Blocking heparanase activity with OGT 2115 or its expression using heparanase siRNA had no effect on the levels of IGFBP-3 in the media of A549 cells (Figure 2A,B). No effects were observed in H1299 cells since they are known to be IGFBP-3-negative [34]. Treatment of cells with either OGT 2115 or with heparanase siRNA decreased the levels of HS in A549 cell media, by ~2.20-fold, and ~1.80-fold in the media of H1299 cells (Figure 2C).

To examine the binding of IGFBP-3 to HS, ELISA plate wells were coated with IGFBP-3 antibodies followed by addition of media obtained from cells untreated or treated with either OGT 2115, control-, or heparanase-siRNA. The amount of HS bound to IGFBP-3 was then detected using anti-HS specific antibodies (Figure 2D). Conversely, HS antibodies were coated in the wells followed by incubation with the same media samples, and then detection of IGFBP-3 was carried out using anti-IGFBP-3 specific antibodies (Figure 2E). In both cases, there was a ~2.0-fold decrease in the binding of HS to IGFBP-3 in the media of A549 cells (Figure 2D,E). As expected, no effects were observed in the media of H1299 cells on HS-IGFBP-3 binding since they are IGFBP-3-negative (Figure 2D,E). An interpretation of these results might be that blocking heparanase activity or expression decreases the levels of soluble HS fragments, consequently leading to decreased binding between IGFBP-3 and HS in the media.

### 3.3. HS Competes with HA for Binding to IGFBP-3 or WT- but Not the Mutant-Peptide

Our data (Figure 2) show that inhibiting heparanase activity or expression resulted in lower levels of HS in the media and reduced amounts of IGFBP-3 bound to HS. We previously found that binding of IGFBP-3, via amino acid residues 215–232 in the C-terminal region of the protein, to HA blocks HA–CD44 interactions reducing A549 cell viability [50]. We also reported that blocking the binding of HA and CD44 with an anti-CD44 antibody (5F12) in combination with IGFBP-3 did not have an additive negative effect on cell viability; thus, suggesting that the cytotoxic effects induced by IGFBP-3 likely arose from a mechanism involving disruption of HA–CD44 interactions [50]. We also showed that the synthetic IGFBP-3 peptide (^215^-KKGFYKKKQCRPSKGRKR-^232^) acted in a manner comparable to that of the full-length IGFBP-3 protein in disrupting HA–CD44 signaling, and that the ^215^-KKGFYKKKQCRPSAGAKR-^232^ mutant peptide (K228AR230A) was unable to bind HA or block HA–CD44 signaling [51,52].

**Figure 3:**
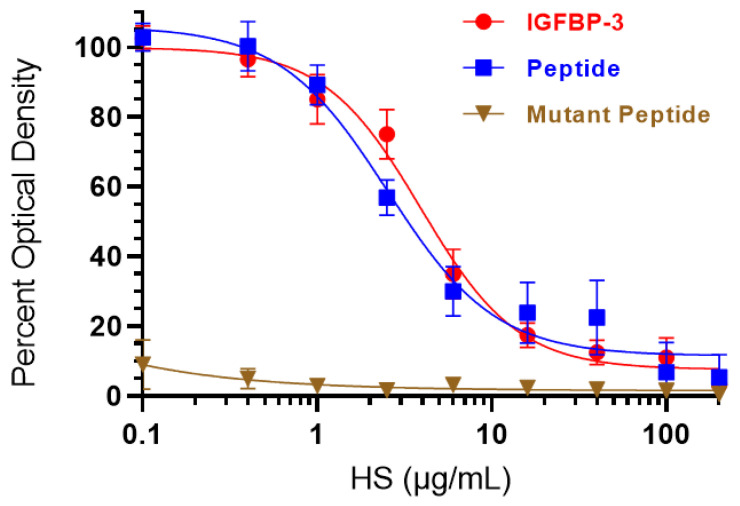
Increasing HS concentrations blocked binding of HA to IGFBP-3 and WT- but not the mutant-peptide. IGFBP-3 protein, WT-, or mutant-peptide (50 nM) were bound to the plate wells. A single concentration of biotinylated-HA (35 µg/mL) was incubated for 1h without or with increasing concentrations of HS and then loaded into the coated wells. The signal was then processed, and the bound biotinylated-HA was then detected as described in the Materials and Methods section. Prior to data analysis, the OD were corrected for non-specific binding by subtracting the mean background absorbance for the negative controls prepared with all components except biotinylated-HA. Optical density measurements (450 nm) were normalized by expressing each point in relation to the best-fitted Emax value for IGFBP-3 (set to 100%). The data were then plotted as a function of increasing HS concentrations. Using the GraphPad Prism 9.4.1 software, the data were analyzed with a nonlinear regression curve fitting approach then expressed as the mean ± S.D. of three independent experiments, each carried out in triplicate.

To examine the effect of HS on the binding of IGFBP-3 protein and peptides to HA, IGFBP-3 protein, WT-, or mutant-peptide were bound to the plate wells (Figure 3). A single concentration of biotinylated-HA was incubated without or with increasing concentrations of HS and then loaded into the coated wells. Bound biotinylated-HA was then detected as described in the Materials and Methods section. HS was effective in blocking the binding of biotinylated-HA to the IGFBP-3 protein with an IC_50_ value of 4.4 ± 0.76 μg/mL and the IGFBP-3 WT-peptide with an IC_50_ value of 3.2 ± 0.55 μg/mL (Figure 3). No effects were found when using the mutant IGFBP-3 peptide which was expected since we have previously reported that the mutant lacks the ability to bind HA [51,52].

### 3.4. HS Abolished the Cytotoxic Effects of IGFBP-3 but Not upon Blocking HA–CD44 Signaling with the 5F12 Antibody

Our results (Figure 3) showed that HS competes with HA for binding IGFBP-3. We have previously shown that the IGFBP-3 protein blocks HA–CD44 signaling and decreases cell viability of the p53-positive A549 cell line more effectively than the p53-negative H1299 cell line [51]. We also reported that blocking HA–CD44 interactions in A549 cells with the 5F12 antibody reduced cell viability to the same extent as that found by the addition of only IGFBP-3 and that IGFBP-3 decreases cell viability by disrupting HA–CD44 interactions [50,51]. While similar trends were observed in the p53-negative cell line, H1299, the inhibition of cell viability was only 10–25% as compared to 50–65% observed in A549 cells suggesting that H1299 might be more resistant to the effects of blocking HA–CD44 with 5F12 or IGFBP-3 [51].

To examine the effect of IGFBP-3 on cell viability in the presence of added HS, in the absence or presence of the CD44 antibody, 5F12, cells were seeded in 96-well plates in 10% FBS-supplemented media. The following day, the cell monolayers were incubated in serum-free medium for 24 h. Fresh serum-free media was then added, and the cells were treated for 72 h, as indicated with the IGFBP-3 protein, the 5F12 antibody, or in combination, without or with increasing HS concentrations. Cell viability was then assessed by the MTT assay as described in the Materials and Methods section.

**Figure 4:**
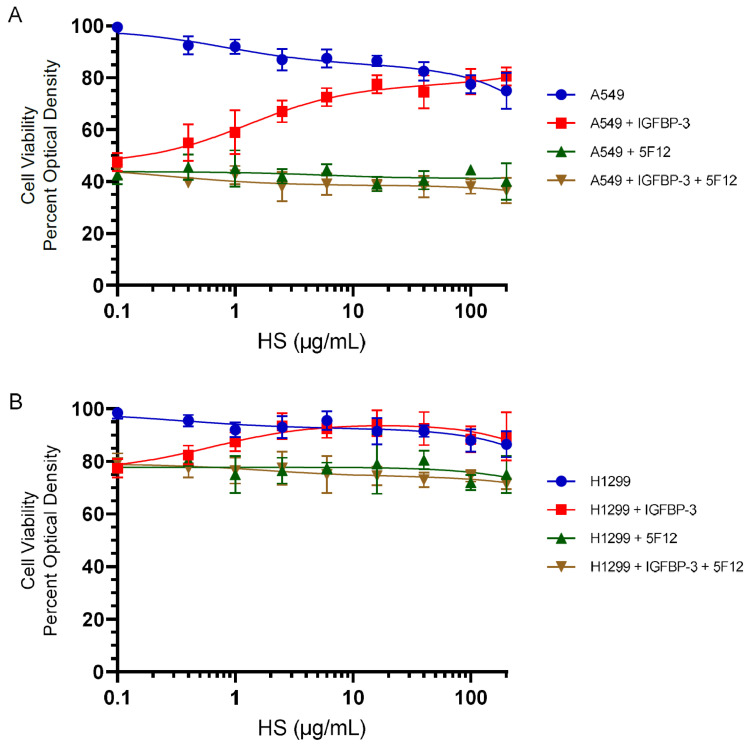
HS blocked the cytotoxic effects of IGFBP-3 but not upon inhibition of HA–CD44 signaling with the 5F12 antibody. Cells were seeded in 96-well plates at 0.2 × 10^5^ cells per well in 10% FBS-supplemented media. The following day, the cell monolayers were incubated in serum-free medium for 24 h. Fresh serum-free media was then added and the cells were treated for 72 h with the IGFBP-3 protein (50 nM), the CD44 antibody (5F12, 5 μg/mL) added either separately or 2 h prior to addition of IGFBP-3, or in combination, without or with increasing HS concentrations. Cell viability of A549 (**A**) and H1299 (**B**) was then assessed by the MTT assay as described in the Materials and Methods section. Optical density measurements (570 nm) were normalized by expressing each point in relation to the best-fitted Emax value of cells without added IGFBP-3 or 5F12 (set to 100%). The data were then plotted as a function of increasing HS concentrations. Using the GraphPad Prism 9.4.1 software, the data were analyzed with a nonlinear regression curve fitting approach then expressed as the mean ± S.D. of three independent experiments, each carried out in triplicate.

Increasing HS concentrations reduced A549 cell viability by ~25% at the highest concentrations used (Figure 4A) while that decrease was ~13.5% in H1299 cells (Figure 4B). Incubation of cells with IGFBP-3, 5F12, or in combination, decreased A549 cell viability by ~55% (Figure 4A) and H1299 cell viability by ~22.5% (Figure 4B). Cell viability was restored upon addition of HS to either cell line treated with only IGFBP-3 but not in cells treated with either 5F12 or a combination of 5F12 and IGFBP-3 (Figure 4). These results might suggest that HS binds IGFBP-3 and blocks its interactions with HA, allowing HA–CD44 signaling to occur, restoring cell survival. These results might also provide further support to the hypothesis that IGFBP-3 operates via disrupting HA–CD44 interactions since blocking this interaction by the 5F12 antibody abolished the ability of HS to alter IGFBP-3-induced effects on cell viability.

### 3.5. Blocking HA–CD44 Signaling Decreases the Levels of Heparanase in the Media of Both Cell Lines and Increases p53 Activity and the Levels of IGFBP-3 in A549 Cell Media

Upon stimulation by various cellular stresses, the p53 tumor-suppressor protein regulates the expression of a vast number of genes involved in blocking cell proliferation, cell-cycle arrest, and induction of senescence and apoptosis [65,66]. The p53 protein was reported to bind a noncanonical p53-binding sequence in the CD44 promoter blocking expression of the CD44 cell-surface protein [26,67]. In lung carcinoma cells, p53 was shown to directly influence the promoter of the CD44 gene, repressing expression of the CD44 protein [67]. In cells lacking functional p53, de-repression of CD44 led to anti-apoptotic and mitogenic effects, tumor cell growth, survival, and metastasis [67]. In hepatocellular carcinoma, CD44 induced AKT activation, which in turn led to phosphorylation and translocation of Mdm2, a negative regulator of p53, to the nucleus, inhibiting the p53 response [68]. High CD44 expression counteracts the p53 tumor-suppressor function promoting tumor growth and survival in different stages of progression, while p53 acts to repress CD44 expression to induce its antiproliferative and apoptotic activities [67,68].

Heparanase expression is regulated by p53, known to directly bind the heparanase gene promoter inhibiting its transcription, while elimination or inhibition of p53 led to increased heparanase gene expression in several cell types [10,63].

The p53 protein is known to act as a transcriptional activator of IGFBP-3 [27,28,44,69,70]. The action of p53 was reported earlier to be blocked by antagonizing IGFBP-3, a p53-response gene that acts to mediate p53-induced apoptosis during serum starvation in cancer cells in an IGF-independent manner [44]. The p53 protein is known to induce IGFBP-3 expression and targeting p53 in lung carcinoma H460 cells for degradation-inhibited apoptosis and enhanced cell growth during serum deprivation compared to untreated control cells [44]. Using an esophageal carcinoma cell line, p53 was increased and stimulated when cells were exposed to IGFBP-3 suggesting an autocrine/paracrine feedback loop between IGFBP-3 and p53 [69].

**Figure 5:**
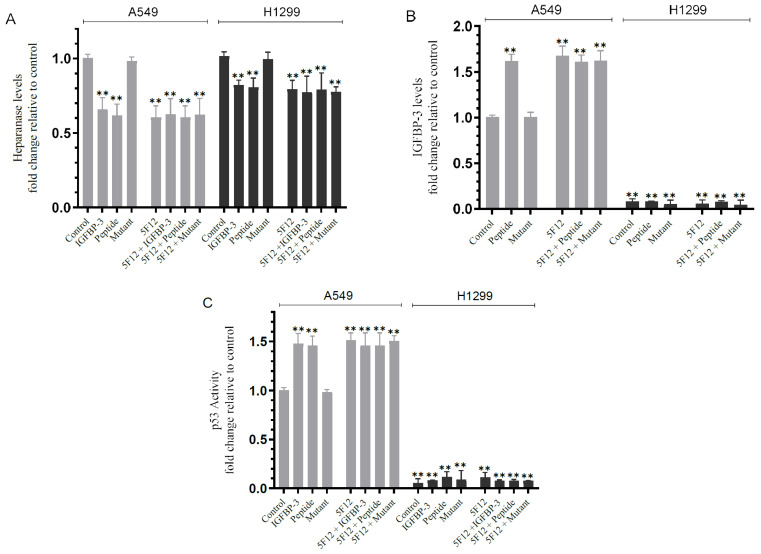
Blocking HA–CD44 signaling decreases the levels of heparanase in the media of both cell lines and increases p53 activity and the levels of IGFBP-3 in A549 cell media. Cells (0.2 × 10^5^) were grown in 10% FBS-supplemented media for 24 h. The following day, the cell monolayers were incubated in serum-free media for 24 h, then treated as indicated for 72 h with 50 nM of IGFBP-3 protein/peptide/mutant, the CD44 antibody (5F12, 5 μg/mL) added either separately or 2 h prior to addition of IGFBP-3/peptide/mutant, or in combination. The levels of heparanase (**A**) and IGFBP-3 (**B**) in the media, using the same amount of protein (3 µL of 600 µg/mL total protein), and the p53 activity (**C**) in cell lysates were measured as described in the Materials and Methods section. The graphs summarize the results expressed as means ± SD (n = 5) using the GraphPad 9.4.1 software. Fold change was calculated relative to the control of each cell line (**A**) or to the A549 control (**B**,**C**). Asterisks indicate a statistically significant difference from the corresponding negative control of each cell line, Mann–Whitney test. Statistical differences between different groups were analyzed by a non-parametric Kruskal–Wallis test. Absence of asterisks indicates no significance, ** *p* < 0.01.

Based on these published reports, we investigated the effect of blocking HA–CD44 signaling on the levels of heparanase, and IGFBP-3 and p53 activation in A549 and H1299 cells (Figure 5). Cells were grown in FBS-supplemented media for 24 h. The following day, the cell monolayers were incubated in serum-free media overnight, then treated as indicated (Figure 5) with 50 nM IGFBP-3 protein, a concentration of added IGFBP-3 chosen to be close to the values we measured previously in the conditioned media of A549 cells [50], IGFBP-3 peptide or mutant peptide (50 nM), the CD44 antibody (5F12, 5 μg/mL), or in combination. The levels of heparanase and IGFBP-3 in the media, and the p53 activity in cell lysates were measured as described in the Materials and Methods section.

Treatment of cells with IGFBP-3 or the peptide decreased the levels of heparanase by ~1.55-fold in A549 cell media and ~1.20-fold in the media of H1299 cells while no effect on those levels was observed when using the mutant peptide (Figure 5A). A comparable decrease in the levels of heparanase (Figure 5A) was obtained by using the 5F12 antibody in the absence or presence of IGFBP-3, peptide or mutant in both cell lines suggesting that the decrease in the levels of heparanase is likely due to the disruption of HA–CD44 signaling.

Treatment of A549 cells with the IGFBP-3 peptide but not the mutant increased the levels of IGFBP-3 in the media by ~1.60-fold, an increase similar to that found upon cell treatment with the 5F12 antibody in the absence or presence of the IGFBP-3 peptide or mutant (Figure 5B). As expected, no effect was found on the levels of IGFBP-3 in H1299 cell media since they are IGFBP-3-negative (Figure 5B).

The p53 activity was enhanced by ~1.50-fold when A549 cells were treated with the IGFBP-3 protein or peptide but not the mutant (Figure 5C). This increase was comparable to that found when A549 cells were incubated with the antibody, 5F12, with or without the IGFBP-3 protein, peptide, or mutant indicating that the increase in the activity of p53 is likely a result of blocking HA–CD44 interactions (Figure 5C). No effects on the activity of p53 were found in H1299 cells since they are known to be p53-null.

### 3.6. Knockdown of p53 Resulted in Increased Heparanase Levels and Reduced IGFBP-3 Levels in A549 Cell Media

**Figure 6:**
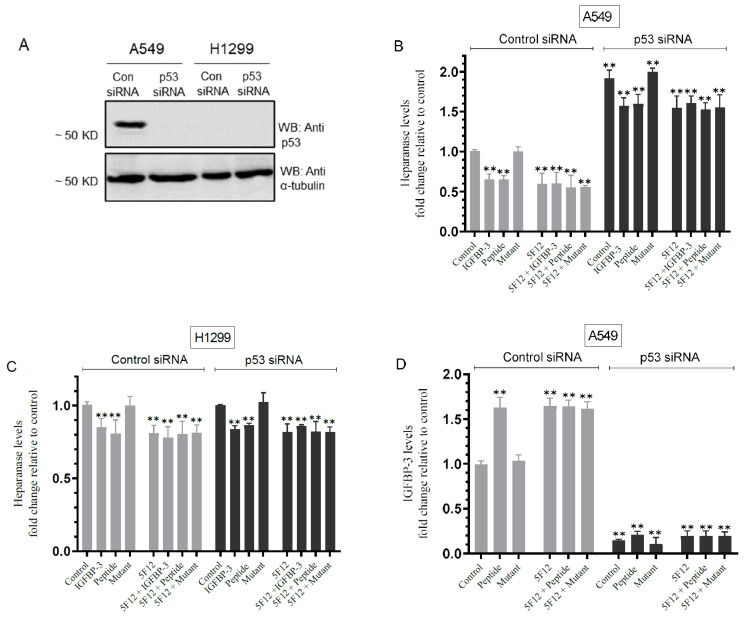
Knockdown of p53 led to increased heparanase levels and decreased IGFBP-3 levels in the media of A549 cells. Cells (0.2 × 10^5^) were grown in 10% FBS-supplemented media for 24 h then serum-starved overnight. The cells were then treated as indicated for 72h with control siRNA or p53 siRNA. The same concentration of total protein (15 µL of 600 µg/mL) of the cell lysates (**A**) was used for Western blotting using the indicated antibodies. As a loading control, anti α-tubulin antibodies were used. Transfected cells were treated with 50 nM of IGFBP-3 protein/peptide/mutant, the CD44 antibody (5F12, 5 μg/mL) added either separately or 2h prior to addition of IGFBP-3/peptide/mutant, or in combination (**B**–**D**). The levels of heparanase (**B**,**C**) and IGFBP-3 (**D**) were measured in the media using the same amount of protein (3 µL of 600 µg/mL total protein) as described in the Materials and Methods section. The graphs summarize the results expressed as means ± SD (*n* = 5) using the GraphPad 9.4.1 software. Fold change was calculated relative to the A549 control siRNA (**B**,**D**) or H1299 control siRNA (**C**). Asterisks indicate a statistically significant difference from the corresponding negative control of each cell line, Mann–Whitney test. Statistical differences between different groups were analyzed by a non-parametric Kruskal–Wallis test. Absence of asterisks indicates no significance, ** *p* < 0.0l.

To investigate the effect of p53 knockdown on the levels of heparanase and IGFBP-3, cells were grown in FBS-supplemented media overnight then serum-starved for 24 h. The cells were then transfected as indicated with control siRNA or p53 siRNA (Figure 6A) with or without treatment using IGFBP-3 protein, peptide, or mutant peptide, the CD44 antibody (5F12), or in combination (Figure 6B–D). The levels of heparanase and IGFBP-3 were then measured in the media as described in the Materials and Methods section.

Treatment of A549 cells with p53 siRNA resulted in a ~1.90-fold increase in the levels of heparanase in the media as compared to cells transfected with control siRNA (Figure 6B); meanwhile, no change in the levels of heparanase was found using H1299 cells since they are p53-negative (Figure 6C). The treatment of A549 cells with control siRNA and IGFBP-3 protein or peptide reduced the levels of heparanase by ~1.55-fold while no effects were found using the mutant IGFBP-3 peptide (Figure 6B). The treatment of A549 cells transfected with control siRNA with 5F12 in the absence or presence of the IGFBP-3 protein, peptide, or mutant led to comparable reductions in the levels of heparanase to those obtained with transfected cells treated with the IGFBP-3 protein or peptide (Figure 6A) and to those in Figure 5A. While the trends were similar, the levels of heparanase were consistently higher in the media of A549 cells transfected with p53 siRNA than those found in the media of A549 cells transfected with control siRNA with the different treatments (Figure 6B). Moreover, the levels of heparanase decreased by only ~1.20-fold when A549 cells transfected with p53 siRNA were treated with IGFBP-3 and/or 5F12 as compared to the more pronounced decrease of ~1.55-fold in those levels in the media of A549 cells transfected with control siRNA and treated under the same conditions (Figure 6A). H1299 cells transfected with either control or p53 siRNA and treated with the IGFBP-3 protein or peptide but not the mutant led to a ~1.15-fold decrease in the levels of heparanase (Figure 6C), results comparable to those obtained in Figure 5A. Similarly, no difference was found in the levels of heparanase in the media of H1299 cells transfected with either control siRNA or p53 siRNA when using the antibody, 5F12, without or with the IGFBP-3 protein, peptide, or mutant (Figure 6C). These results show that the differences in the levels of heparanase upon transfection of A549 cells with p53 siRNA were more similar to those obtained for H1299 transfectants as compared to A549 cells transfected with control siRNA (Figure 6B,C) and suggest that p53 acts to suppress the levels of heparanase in the media of A549 cells.

Comparable to the results obtained in Figure 5B, the levels of IGFBP-3 increased by ~1.65-fold in A549 cells transfected with control siRNA and treated with the IGFBP-3 peptide but not the mutant or upon treatment of the control siRNA transfectants with the 5F12 antibody without or with the IGFBP-3 peptide or mutant (Figure 6D). Transfection of A549 cells with p53 siRNA reduced the levels of IGFBP-3 in the media by ~7.00-fold as compared to A549 cells transfected with control siRNA (Figure 6D). No significant effects were observed with any of the treatments on those levels suggesting that p53 knockdown decreases the levels of IGFBP-3 in A549 cell media.

### 3.7. Knockdown of IGFBP-3 in A549 Cells Inhibited p53 Activity and Increased Heparanase Levels in the Media

**Figure 7:**
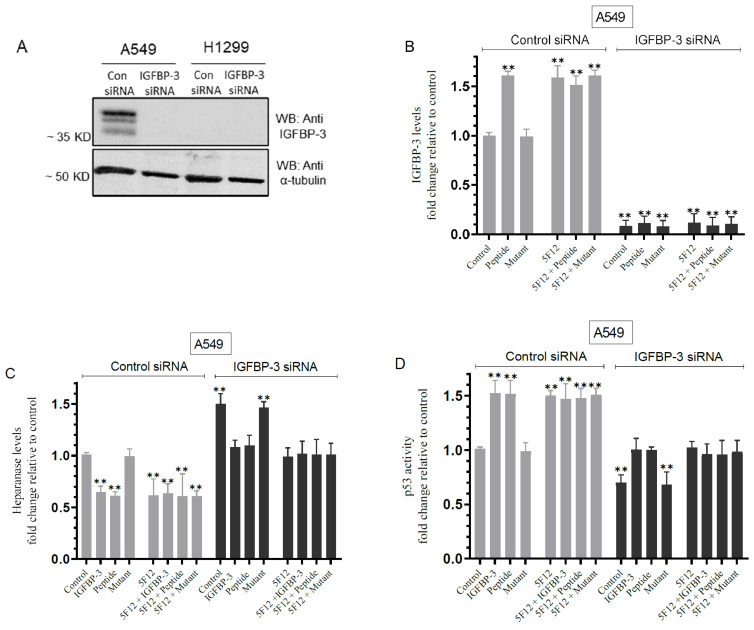
Knockdown of IGFBP-3 led to increased heparanase levels in the media of A549 cells and decreased p53 activity. Cells (0.2 × 10^5^) were grown in 10% FBS-supplemented media for 24 h then serum starved overnight. The cells were then treated as indicated for 72 h with control siRNA or IGFBP-3 siRNA. The same concentration of total protein (15 µL of 600 µg/mL) of the cell lysates (**A**) was used for Western blotting using the indicated antibodies. As a loading control, anti α-tubulin antibodies were used. Cell transfectants were treated without or with 50 nM IGFBP-3 protein/peptide/mutant, the CD44 antibody (5F12, 5 μg/mL) added either separately or 2 h prior to addition of IGFBP-3 protein/peptide/mutant, or in combination (**B**–**D**). The media levels of IGFBP-3 (**B**) and heparanase (**C**) using the same amount of protein (3 µL of 600 µg/mL total protein), and the p53 activity in cell lysates (**D**) were measured as described in the Materials and Methods section. The graphs summarize the results expressed as means ± SD (*n* = 5) using the GraphPad 9.4.1 software. Fold change was calculated relative to the A549 control siRNA transfectants (Control). Asterisks indicate a statistically significant difference from the corresponding A549 negative control, Mann–Whitney test. Statistical differences between different groups were analyzed by a non-parametric Kruskal–Wallis test. Absence of asterisks indicates no significance, ** *p* < 0.0l.

We next investigated the effect of IGFBP-3 knockdown on heparanase and p53 (Figure 7). Cells were grown in 10% FBS-supplemented media for 24 h then serum-starved overnight. The cells were then treated as indicated with control siRNA or IGFBP-3 siRNA, without or with IGFBP-3 protein, peptide, or mutant peptide, the CD44 antibody, or in combination. The media levels of IGFBP-3 and heparanase along with the p53 activity in the cell lysates were measured as described in the Materials and Methods section.

A549 cell treatment with IGFBP-3 siRNA effectively reduced the levels of the protein (Figure 7A,B). Consistent with the results shown in Figure 5 and Figure 6, treatment of A549 cells with the IGFBP-3 peptide, but not the mutant, or 5F12 in the absence or presence of peptide or mutant led to a ~1.60-fold increase in the levels of IGFBP-3 in the media (Figure 7B) and a ~1.55-fold decrease in the levels of heparanase in the media (Figure 7C). Transfection of A549 cells with IGFBP-3 siRNA led to a ~1.50-fold increase in heparanase levels in the media (Figure 7C). Treatment of A549 cells transfected with IGFBP-3 siRNA with the IGFBP-3 protein, peptide, 5F12, or in combination, decreased the levels of heparanase by ~1.45-fold (Figure 7C). In contrast to the results obtained with heparanase (Figure 7C), p53 activity was increased by ~1.50-fold in A549 cells transfected with control siRNA and treated with either the IGFBP-3 protein, peptide, 5F12, or in combination (Figure 7D). Transfection of A549 cells with IGFBP-3 siRNA decreased the activity of p53 by ~1.45-fold and treatment of those cells with the IGFBP-3 protein, peptide, 5F12, or in combination increased the activity of p53 by ~1.45-fold (Figure 7D).

## 4. Discussion

In this study, we examined the role of heparanase and IGFBP-3 in regulating NSCLC cell survival. Previous work has shown that the tumor suppressor, p53, binds directly to the heparanase gene promoter inhibiting its transcription while, conversely, elimination or inhibition of p53 led to increased heparanase gene expression and enzymatic activity in several cell types [10,63]. We found that H1299 cells known to be p53-null with no expression of IGFBP-3 had higher heparanase levels and activity and higher levels of HS in the media compared to the media of A549 cells (Figure 1).

The mechanistic interplay between the GAGs and IGFBP-3 in regulating a variety of players in carbohydrate signaling, and the consequent impact of that interplay on cell survival and death, are important but not fully understood. Mature IGFBP-3 is known to bind GAGs including HS via its 18-basic amino acid motif in the C-terminal domain (^215^-KKGFYKKKQCRPSKGRKR-^232^) [27,28,46,47,48]. Protein–HS interactions are predominantly driven by charge–charge interactions between the polysaccharide’s anionic carboxylate and/or sulfate groups and the protein’s basic amino acids [4,6]. Proteins or peptides with positively charged amino acid residues may serve as HS-dependent treatments for cancer therapy since they are able to antagonize HS chains by binding to the negatively sulfated groups [6,6,46]. Inhibiting heparanase activity with OGT 2115 or its expression using siRNA (Figure 2) had no effect on the levels of IGFBP-3 in the media of A549 cells (Figure 2A,B), decreased HS level in the media (Figure 2C), and reduced the amount of IGFBP-3 bound to HS (Figure 2D,E). Lower levels of cell-surface HS have been found to correlate with a high metastatic capacity of a variety of tumors [6]. No effects were observed in H1299 cells since they are known to be IGFBP-3-negative [34]. These results might indicate that blocking heparanase activity or expression reduces the levels of soluble HS fragments, consequently leading to decreased interactions between IGFBP-3 and HS in the media.

IGFBP-3 has both IGF-dependent and independent antiproliferative effects [44,70]. We previously reported that the binding of IGFBP-3, via amino acid residues 215–232 in the C-terminal region of the protein, to HA inhibits HA–CD44 interactions decreasing A549 cell viability [50]. We also showed that blocking the binding of HA and CD44 using an anti-CD44 antibody (5F12) in combination with IGFBP-3, did not result in an additive negative effect on cell viability, indicating that the cytotoxic effects induced by IGFBP-3 likely result from a mechanism involving disruption of HA–CD44 interactions [50]. We also found that the synthetic IGFBP-3 peptide (^215^-KKGFYKKKQCRPSKGRKR-^232^) acted in a manner comparable to that of the full-length IGFBP-3 protein in blocking HA–CD44 signaling, and that the ^215^-KKGFYKKKQCRPSAGAKR-^232^ mutant peptide (K228AR230A) failed at binding HA or blocking HA–CD44 signaling [51,52]. In this study, we found (Figure 3) that HS competes with HA for binding to the IGFBP-3 or WT-peptide but not the mutant-peptide.

Previously, we showed that IGFBP-3 blocks HA–CD44 signaling and decreases the cell viability of the p53-positive A549 cell line to a greater extent than the p53-negative H1299 cell line; thus, suggesting that H1299 might be more resistant to the effects of blocking HA–CD44 with 5F12 or IGFBP-3 [51]. We also reported that IGFBP-3 decreases cell viability by disrupting HA–CD44 interactions [50,51]. Here, we found that HS abolished the cytotoxic effects of IGFBP-3 but not upon blocking HA–CD44 signaling with the 5F12 antibody (Figure 4). Cell viability was restored upon addition of HS to either A549 or H1299 cells treated with only IGFBP-3 but not in cells treated with either 5F12 or a combination of 5F12 and IGFBP-3 (Figure 4). These findings might suggest that the binding of HS to IGFBP-3 blocks the protein’s interactions with HA, allowing HA–CD44 signaling to occur, restoring cell survival. These results support the hypothesis that IGFBP-3 operates via disrupting HA–CD44 interactions since using the CD44 antibody, 5F12, to block these interactions abolished the ability of HS to alter IGFBP-3 induced effects on cell viability (Figure 4).

Blocking HA–CD44 signaling decreased the levels of heparanase in the media of both A549 and H1299 cell lines and increased p53 activity and the levels of IGFBP-3 in A549 cell media (Figure 5). High CD44 expression is known to counteract p53 tumor-suppressor function increasing tumor cell growth and survival in different stages of progression while p53 has been shown to act as a repressor of CD44 expression to induce its antiproliferative and apoptotic activities [67,68]. The increase in the level and abundance of IGFBP-3 in the conditioned media of A549 cells is likely because the IGFBP-3 gene is known to be a p53-regulated target gene and the protein is predominantly secreted [71]. Expression of heparanase is regulated by p53, that directly binds the heparanase gene promoter blocking its transcription, while elimination or inhibition of p53 led to increased heparanase gene expression in a variety of cell types [10,63]. The decreased heparanase levels in the media of the p53-null H1299 cells might be due to the effect on other mechanisms involving, for example, the early growth response gene 1 (EGR1) that binds the heparanase promoter, regulating its transcription in tumor cells, and acts as a key regulator of inducible heparanase transcription in certain cancers [12]. The p53 protein is known to act as a transcriptional activator of IGFBP-3 inducing its expression [27,28,44,69,70]. The action of p53 is blocked by antagonizing IGFBP-3, a p53-response gene, during serum-starvation in cancer cells in an IGF-independent manner [44]. Using an esophageal carcinoma cell line, p53 was increased and activated upon cell exposure to IGFBP-3 suggesting an autocrine/paracrine feedback loop between IGFBP-3 and p53 [69].

**Figure 8:**
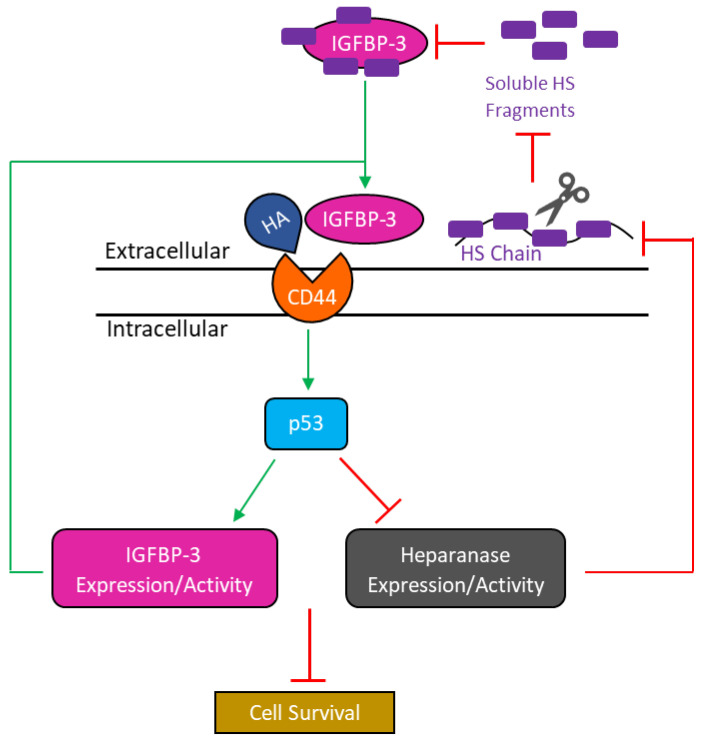
Representation of the main hypothesis and findings from this study. Binding of IGFBP-3 to HA blocks HA–CD44 signaling leading to p53 activation which in turn results in increased IGFBP-3 levels. IGFBP-3 is now able to continue disrupting HA–CD44 signaling further increasing its own levels. Increased p53 activation resulting from disruption of HA–CD44 signaling by IGFBP-3 also leads to decreased heparanase levels and activity, blocking the enzyme’s ability to cleave cell-surface HS chains, decreasing soluble HS fragments, and formation of the IGFBP-3-HS complex. IGFBP-3 not bound to HS is now able to bind HA disrupting HA–CD44 signaling decreasing cell survival. Green lines/arrows indicate activation while red lines indicate inhibition.

We previously found that IGFBP-3 blocks HA–CD44 signaling via a mechanism that depends on both p53 and AChE [51]. In this study, our data show that knockdown of p53 led to increased heparanase levels and reduced IGFBP-3 levels in A549 cell media (Figure 6). We also show that knockdown of IGFBP-3 in A549 cells blocked p53 activity and increased heparanase levels in the media (Figure 7). Based on our findings, we propose a model summarizing the main hypothesis and key points of this study (Figure 8). In this study, we used A549 (p53-positive) and H1299 (p53-null) cells to examine the effect of p53 on IGFBP-3 and heparanase signaling in NSCLC cells. It is widely known, however, that *TP53* differs from most other tumor-suppressor genes, in that it is primarily altered by missense as opposed to truncating mutations [72,73,74,75,76]. Frameshift and nonsense mutations generally result in loss of protein expression or a truncated protein, whereas missense mutations can cause overexpression of the p53 mutant protein or have no effect on translation [73,75,76,77,78]. Mutations of p53 are known to lead to different functions ranging from total loss-of-function to gain-of-function [76,79]. Previous meta-analysis found that mutations in *TP53* led to a shorter survival rate in patients with adenocarcinoma and those in certain stages of NSCLC [80]. Compared to wild-type *TP53*, the mutated gene in NSCLC was shown to lead to increased tumor progression and greater resistance to chemotherapy [81,82]. Therefore, examination of the effects of IGFBP-3 and heparanase in A549 and H1299 cells harboring different missense mutations of *TP53* merits further examination.

## Data Availability

Not applicable.
